# Influence of Replacement Part of Starch with Inulin on the Rheological Properties of Pastes and Gels Based on Potato Starch

**DOI:** 10.1155/2020/7642041

**Published:** 2020-08-26

**Authors:** Mariusz Witczak, Zofia Smółka, Teresa Witczak, Anna Stępień, Agata Bednarz

**Affiliations:** Department of Engineering and Machinery for Food Industry, University of Agriculture in Krakow, Balicka 122 Str., 30-149 Kraków, Poland

## Abstract

The objective of the present study was to determine the influence of replacement part of starch with inulin on the rheological characteristics of pastes and gels obtained on the basis of potato starch. Replacement of the starch by inulin varied from 0 to 40%. Flow curves for pastes and gels were determined, and the viscoelastic properties were characterized using dynamic tests and creep and recovery tests. It was determined that the replacement of part of potato starch with inulin significantly modifies rheological properties of starch pastes and gels, weakening their structure. With the increasing amount of inulin, an increase of viscous properties was becoming more apparent. Moreover, an irregular influence of inulin addition on the parameters of rheological characteristics was determined. Initially, the differences were minor, and the differences at the lowest addition were typically statistically insignificant, followed by strong increase with local restrictions to structural weakening.

## 1. Introduction

Inulin and starch are among the most widely distributed biopolymers, forming a reserve material of numerous plant species. Inulin has exhibited many interesting functional and health-promoting properties [1]. Its structure is based on fructose residues connected by *β*(2,1)-glycosidic bonds, ended with singular glucose molecules. Due to its properties depending on the polymerization degree and the nature of the bonds, attempts have been made to use the compound as a component of numerous food products [1, 2].

Natural starch occurs in the form of granules mostly in seeds, roots, and tubers, as well as stems, leaves, and fruit. Starch granules differ in size and shape, primarily depending on the botanical origin. In seeds, starch occurs in a partially crystalline form. The crystallinity degree depends on the botanical origin (amylose to amylopectin ratio), and it influences certain parameters of starch to a large degree. The botanical origin further significantly influences the additional substances occurring in starch, which can be described as concomitant. Their type and amount determines its physicochemical and functional properties [3].

Since inulin is considered a substance that can have a beneficial effect on health and at the same time guarantee a sufficiently high level of sensory properties of food products, there is a lot of research on its application in food. In many cases, inulin occurs in combination with other polysaccharides, among other starch of various botanical origins (potato, corn, tapioca, and rice). The work on gluten-free bread supplementation with inulin can be quoted here [4–6]; combinations of a multicomponent system containing, among others, starch and inulin as wall materials in the process of microencapsulation of rosemary essential oil were studied by de Barros Fernandes et al. [7]. Inulin was also used as a component of spaghetti [8], the effect of inulin in the presence of potato starch as culture media for *Lactobacillus casei* [9] and in the presence of modified starch as a ketchup component [10]. Inulin and starch have also been tested for use in the production of bioactive symbiotic edible film [11].

Despite the fact that inulin is highly commonly used in food products in combination with starch and nonstarch hydrocolloids, information on their interactions is limited. Here, studies of [12, 13] can be mentioned devoted to interactions of inulin with maize starch and studies on the interaction of inulin with potato, wheat, and rice starch [14–17]. With the exception for the studies of [12, 13], the remaining studies concern the degree of polymerization and analyse properties of inulin selected values of additives. On the other hand, a large number of studies focus on the using of inulin in practical systems such as meat, bakery, dairy, flour, chocolate, and other products [18, 19]. However, the image presented in those studies is not unambiguous. In some cases, inulin addition results in increasing values of the parameters of the rheological characteristics, and in other, their reduction, whereas in other instances, depending on the concentration, they first increase then decrease. Here, the presence of other components has significant impact on the nature of inulin-starch interactions, and it does not enable distinction of direct interactions between the analysed biopolymers in such systems; despite the fact that they have significant impact on the final properties of products, they are contained in. Although these interactions depend on the mutual relationship between these polymers, few studies have analysed the properties of such systems in a wider scope for the change of concentration of both biopolymers. Thus, the present study aimed at determining the influence of inulin on rheological properties of starch-based pastes and gels in a wider scope of the change of the starch/inulin ratio.

## 2. Materials and Methods

### 2.1. Materials

Potato starch (ZETPEZET, Poland) and inulin Frutafit®IQ (Sensus, Roosedaal, The Netherlands) with DP 8-12 [20, 21] were used in the study. The following were used as model systems: 5% pastes and gels were subject to analysis, in which potato starch was replaced with inulin (0%, 5%, 10%, 15%, 20%, 25%, 30%, 35%, and 40%). The range of inulin amounts was based on the doses used with starch systems, extending slightly to make rheological parameters dependent on concentration. Weighted portions of distilled water were heated to 40°C; then, weighed amounts of inulin and starch were gradually added with continuous stirring. The obtained suspension was mixed for 15 min at 40°C on the magnetic stirrer working at 150 rpm. Next, the samples were placed in a water bath and were pasted (95°C for 30 min) continuously with a mechanical stirrer (IKA Eurostar Power Visco, Germany) at 500 rpm. For measurements of flow curves at 50°C, the pasted samples were placed in a rheometer system and cooled to measurement temperature. For measurements at 25°C, the samples were cooled in an ultrathermostat for 1 hour to a temperature of 25°C and then transferred to the rheometer system.

### 2.2. Methods

Rheological properties were characterized using a rheometer MARS II (Thermo Haake, Germany) equipped with the cone-plate system (diameter 35 mm, gap 0.105 mm). The samples were placed in the rheometer measuring system, the excess of the sample was removed, and the edges were covered with paraffin oil. The sample was left for 5 minutes (pastes) and 15 minutes (gels) to relax stress and stabilize the temperature, and then, the measurements were performed.

The paste was characterized at 50°C by determining flow curves at increasing and decreasing shear rate in the range 0.1-200 s^−1^ (180 s up and 180 s down). Based on the experimental data, power-law model parameters were determined [16]:

(1) $$\begin{array}{c} \tau =K∙{\dot{\gamma }}^{n}, \end{array}$$

where *τ* is the shear stress (Pa), *K* the consistency coefficient (Pa s^*n*^), $\dot{\gamma }$ the shear rate (s^−1^), and *n* the flow behaviour index.

Gels were characterized at 25°C. Analogously to pastes, flow curves were determined at increasing and decreasing shear rate in the range 0.1-200 s^−1^ (180 s up and 180 s down), and mechanical spectra and creep and recovery tests within the range of linear viscoelasticity were performed.

The range of linear viscoelasticity was determined by verifying the dependence of storage *G*′ and loss *G*^″^ moduli on the applied stress in the range of 0.1-100 Pa at a fixed frequency of 1 Hz.

Mechanical spectra were determined in the range of linear viscoelasticity at constant strain amplitude of 5% in the angular frequency of 1-100 rad s^−1^. The experimental data were fitted with power-law equations [22]:

(2) $$\begin{array}{c} G^{\prime }\left(\omega \right)=K^{\prime }∙\omega^{n^{\prime }}, \end{array}$$

(3) $$\begin{array}{c} G^{\prime \prime }\left(\omega \right)=K^{\prime \prime }∙\omega^{n^{\prime \prime }}, \end{array}$$

where *G*′ is the conservative modulus (Pa), *G*^″^ the loss modulus (Pa), and *ω* the angular velocity (rad s^−1^), while *K*′, *K*^″^, *n*′, and *n*^″^ are experimentally determined constants.

Creep and recovery tests were performed at fixed stress *σ*_0_ = 1 Pa in the range of proportionality of strain to stress. The creep phase continued for 150 s, and recovery 300 s. The experimental data were fitted by Burger's model:

(4) $$\begin{array}{c} J\left(t\right)=J_{0}+\frac{t}{\eta_{0}}+J_{1}\cdot \left(1-\exp^{-t/\lambda_{\text{ret}}}\right)\;\text{for }t<t_{1}, \end{array}$$

(5) $$\begin{array}{c} J\left(t\right)=\frac{t_{1}}{\eta_{0}}-J_{1}\cdot \left(1-\exp^{t_{1}/\lambda_{\text{ret}}}\right)\cdot \exp^{-t/\lambda_{\text{ret}}}\;\text{for }t>t_{1}, \end{array}$$

where *J* is the compliance (Pa^−1^), *J*_0_ is the instantaneous compliance, *J*_1_ is the retardation compliance (Pa^−1^), *η*_0_ is zero shear viscosity (Pa∙s), *λ*_ret_ is the retardation time (s), and *t*_1_ is the time after which stress was removed (s).

Calculations were performed using the Marquardt-Levenberg method with the use of the Statistica 12.0 software (StatSoft Inc., USA).

### 2.3. Statistical Analyses

In order to establish the statistical differences between means, the data were treated by one-factor analysis of variance, and the averages were compared with the Duncan test at significance level 0.05. All calculations were performed with the statistical software package Statistica 9.0 (StatSoft Inc., USA).

## 3. Results and Discussion

### 3.1. Flow Behaviour of Starch Paste

**Figure 1:**
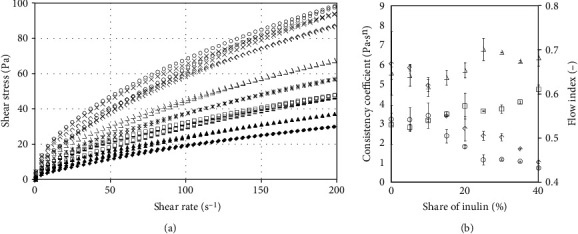
Influence of inulin addition on flow characteristics of pastes at temperature 50°C: (a) flow curves: ◯ 0%, × 5%, ◊ 10%, ∆ 15%, ∗ 20%, □ 25%, − 30%, ▲ 35%, and ♦ 40%; (b) dependency of power-law model parameters on the contribution of inulin: curve at increasing shear rate: ◯ *K*, ∆ *n*; curve at decreasing shear rate: ◊ *K*, □ *n*.

Figure 1 demonstrates flow curves at 50°C of potato starch pastes and pastes, in which part of starch was replaced by inulin and the reliance of power-law model constants on the inulin contribution. Characteristics of starch pasting in aqueous systems depend on physical and chemical properties of starch granules [3]. Nonstarch hydrocolloids also have a strong impact on the behaviour of the starch during pasting [23], primarily through restriction of the phenomenon of starch granule amorphous region hydratation [22]. As a whole, the presence of nonstarch components in the system of pasting and gelatinizing starch leads to significant modifications of the formed structure and as a result its strengthening or weakening. In the tested case, the course of flow curves was typical for shear thinning non-Newtonian fluids. However, analysis of the curve courses with increasing and decreasing shearing rate, due to the increase of shear stress values, suggests the occurrence of the phenomenon of antithixotropy. But taking into account the values of the hysteresis loop surface area at 25°C (positive) and the fact that in the starch gruel at 50°C the process of formation/reinforcement of the produced gel structure occurs and, additionally, the negative surface area values obtained (being a quantitative measure of antithixotropy) are characterized by high variability, the authors believe that these values are the effect of the gel structure reinforcement, i.e., it is not due to the shear reinforcement (antithixotropy), but to the reinforcement of the structure as a result of the fast (during tests) gelation process.

This variability may be also result of the presence of the inulin in the system and its participation in the structure production process. As determined in earlier studies, inulin gel formation is a combination of the crystallization process and formation of three-dimensional network by the crystals, and the final properties of gel depend on the mutual ordering of the crystals. The crystallization process and thus the final properties of the system are difficult to control as well [24]. However, it should be noted that the increase of inulin content and thus decrease of starch concentration resulted in a decrease of the shear stress values, and eventually in weakening of the structure. However, the reduction of stress value was not regular. At a small addition of inulin (5-15%), the reduction of stress value was minor, followed by considerable intensification. In the range 30-35%, the decreasing was limited and then intensified again. The irregular influence of inulin on the viscosity of the systems can be found in the paper of [25]. The authors determined an increase of viscosity during pasting, but it was irregular. In turn, a decrease of final viscosity in experiments using RVA was obtained in the studies of [15, 16, 26]. This suggests various mechanisms of the impact of inulin presence on the paste properties depending on its contribution to the suspension subject to pasting and gelling.

Parameters of the power-law model used for the description of the experimental data are listed in Table 1. With the increasing inulin content in the tested samples, the consistency index *K* is reduced, both at increasing (3.20-0.72 Pa s^*n*^) and decreasing shear rate (6.06-1.03 Pa s^*n*^). In line with the description for shear stress presented above, higher *K* values at decreasing shear rate and negative values of the hysteresis loop surface area were obtained. Change of the consistency indices values (Figure 1) was irregular, similarly to stress, initially exhibiting a minor decrease, followed by a rapid value decrease. Considerable variability was observed for the hysteresis loop surface area, and the differences were statistically insignificant, despite considerable differences in mean values. This indicates high dynamics of structural changes occurring within the tested paste, associated with structure formation during pasting and gelation. Values of the flow behaviour index at increased rate were alternately increasing and decreasing. This suggests considerable variability of the internal structure of the obtained gels. However, it should be noted that in the range up to 25% of inulin contribution, no statistically significant changes were observed, and only the highest values of addition resulted in increased *n* value. Structure destruction resulted in gradual changes of the flow behaviour index, from the lowest for 0% to highest for 40% share of inulin. However, also in this case, the changes were not regular.

Temperature and heating time are among the basic parameters influencing rheological properties of inulin gels [24]. In line with the study results, heating at lower temperatures (20-40°C) leads to formation of a firm gel, whereas temperature increasing causes deteriorated gel properties as the effect of reduction of the amount of crystallization nuclei [27]. According to various authors, inulin forms gel structure on the basis of small crystallites [28], and dissolving crystallites at temperature above 80°C results inhibition of gelatinization process [27, 28]. Results of the presented study confirm the previous conclusions. Samples were prepared in line with methodology for starch, at over 80°C, which most likely led to restriction of the contribution of inulin in the structure forming process, and the simultaneous change (removal) of a portion of starch replaced with inulin led to the reduction of the viscosity of the tested systems due to the lack of structure produced by inulin.

### 3.2. Flow Behaviour of Starch Gel

**Figure 2:**
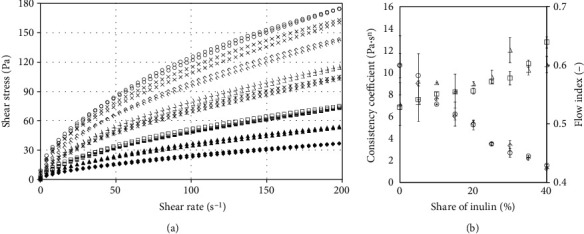
Influence of inulin addition on flow characteristics of gels at temperature 25°C: (a) flow curves: ◯ 0%, × 5%, ◊ 10%, ∆ 15%, ∗ 20%, □ 25%, − 30%, ▲ 35%, and ♦ 40%; (b) dependency of power-law model parameters on the contribution of inulin: curve at increasing shear rate: ◯ *K*, ∆ *n*; curve at decreasing shear rate: ◊ *K*, □ *n*.

Figure 2 presents the course of flow curves, and Table 2 lists the parameters of power-law equation describing the rheological properties of the tested systems at 25°C. Also in this case, the value of shear stress decreased with the increase of inulin content. The decrease of the shear stress value was irregular, similarly to pastes. In this case (25°C), the value of the hysteresis loop surface area was positive, which indicates the occurrence of thixotropy. The results were characterized by high variability (the probable causes are discussed above), yet in this case, the statistical analysis demonstrated that the means differ statistically significantly, although the differences between the means were lower than that for pastes. The consistency coefficient value decreased with the increasing of inulin concentration. However, contrary to paste (flow curves at 50°C temperature), the decrease was much more regular. Only at above 30% inulin contribution, the *K* decreased at a significantly slower rate. In the range from 30%, the decrease was close to linear relative to the inulin concentration. Moreover, it should be noted that in this case, the influence of inulin concentration on the consistency coefficient for the increasing and decreasing curve had a similar course. The flow behaviour index for the primary structure increased initially (but the changes were not considerable), and then it decreased. In turn for the destroyed structure (curve downwards), the flow behaviour index increased gradually, attaining the highest value for the highest inulin addition.

Earlier studies [29] determined the increase of viscosity for emulsions with addition of inulin which according to authors could have stemmed from the increase of total solid content or the occurrence of interaction between oligosaccharide and polysaccharide. As stated by the authors, the change was gradual from 2.5 to 7.5%, and in the range between 7.5 and 10%, a rapid increase of viscosity was observed. In turn, a decrease of *K* and *n* after replacement of a part of rice starch with short and medium chain inulin was determined in the study of [26]. On the other hand, Kou et al. [14], who examined the influence of inulin on the properties of gels obtained on the basis of wheat starch determined variable influence, depending on inulin DP. This indicates the variable mechanisms of creating structure and the issues associated with the control of the structure forming process by inulin or variable interactions between inulin and starch, depending on the concentration of inulin and/or relative ratio between inulin and starch. This is further confirmed by the results of the *K* value obtained in the study of [2]. Despite the considerable differences relative to control sample, depending on the type of inulin, no statistically significant differences were found, which stemmed from the considerable variability of the obtained values. On the other hand, in the study of [30], an increase of the consistency coefficient was obtained for three types of inulin added to milk-sour cherry juice mixture. According to the authors, the cause of the increasing viscosity could be both: the increase of the chain length and the process associated with crystallization and aggregation.

Taking into account the cited works and the results of this work, it should be stated that for systems prepared according to the starch methodology, there is a weakening of the structure due to the removal of starch and the lack of structure produced by inulin, while the local limitations of viscosity decrease result from inulin-starch and inulin-inulin interactions.

### 3.3. Viscoelastic Properties of Starch Gel

**Figure 3:**
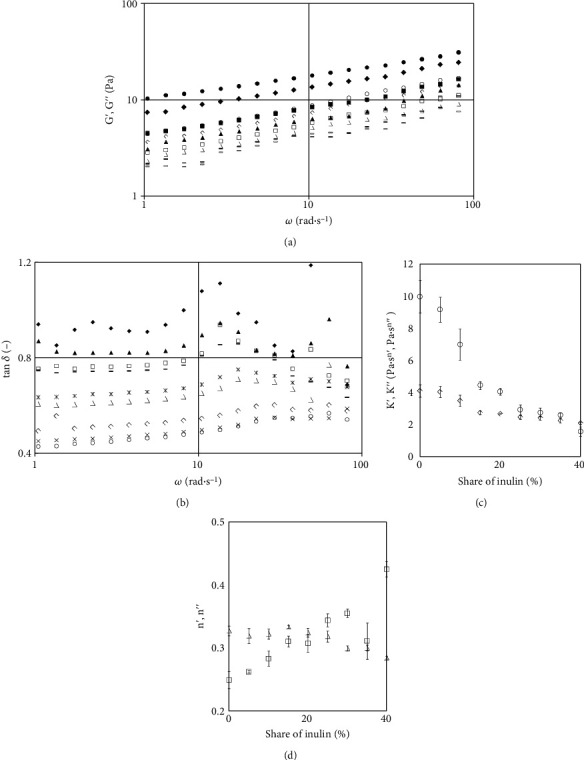
Mechanical spectra, tangent of the phase shift angle, and dependency of power-law model parameters on inulin contribution: (a) mechanical spectra (*G*′ filled symbols, *G*^″^ empty symbols): ◯, • 0%; ◊, ♦ 10%; □, ▪, 20%; ∆, ▲ 30%; and − , □ 40%. (b) Tangent of the phase shift angle: ◯ 0%, × 5%, ◊ 10%, ∆ 15%, ∗ 20% □ 25%, − 30%, ▲ 35%, and ♦ 40%. (c) Dependency of *K*′ and *K*^″^ on inulin contribution: *K*′, ◊ *K*^″^. (d) Dependency of *n*′ and *n*^″^ on inulin contribution: □ *n*′, ∆ *n*^″^.

Figure 3 presents the mechanical spectra of tested samples, dependency of phase shift angle tangent of angular frequency, and dependency of power-law model parameters of the contribution of inulin in a sample. For control sample (without inulin), the *G*′ values were higher than *G*^″^ (tan *δ* < 1) and increased with the increase of the angular frequency values. Tan *δ* values and the course of the relationships indicate a significant advantage of elastic properties over viscous properties and enable the statement that the sample has the character of a strong gel. The obtained results comply with the results of the preexisting research on other products such as ketchup, kefir, yoghurt, and gluten-free bread dough [4, 10, 16, 22, 31, 32]. Addition of inulin results in reduction of the moduli values, maintaining of the course of moduli dependencies of angular frequency, and at the same time causing increase of the value of phase shift angle and its considerable variability in the function of angular speed. Increase of tan *δ* value over 1 was obtained for the highest addition of inulin. This means that the tested systems maintain gel structure with its simultaneous strong weakening and significant increase of the share of viscous properties of the tested systems.

Earlier studies [13] demonstrated that addition of the inulin results in reduction of the value of the loss and storage moduli. Decrease of the value of moduli was also obtained in the study of [33]. The level of decrease depended on the inulin content, and stronger decrease was determined for the preparation with its lower content. On the other hand, Chetachukwu et al. [34] obtained moduli increase up to 15% inulin addition, followed by decrease at 20%. They determined the 15% addition as the critical value, and changes in the rheological characteristics were largely assigned to the inulin-protein interactions and modifications of the protein-protein interactions. Similarly, Kou et al. [14] who investigated the influence of the inulin addition on the properties of gels based on wheat starch determined change of the moduli values, increase to a certain range, depending on the chain length, and, in others, a decrease. The changes were assigned to the interference of inulin in the three-dimensional network created by amylose. On the other hand, a variable influence was obtained with medium chain length and the DP range (2-60). Initially, a minor increase, followed by decrease and at high concentration (15%) *G*′ and *G*^″^ of inulin, starch gel were significantly increased, which according to authors indicates that inulin molecules could have interacted with each other to form a weakly elastic gel. The influence was also variable with addition of a long-chain inulin; at lower concentrations, an increase of the moduli was obtained, and at higher concentrations, a decrease, obtaining lower values than for control sample. In the study of [26], the addition of the inulin did not change the sample characteristics, as all exhibited weak gel-like behaviour. Authors did not determine significant influence of the replacement of part of the rice starch with short chain inulin on the moduli values, whereas replacement with medium and long-chain inulin increased moduli values considerably. In turn, Alvarez-Sabatel et al. [35] determined increased stability of emulsion with 6 and 12% addition of inulin. Structural changes during storage were demonstrated in the study. Authors determined that the dependency of structure development on time results from the time required for inulin gel development. Similarly, increasing of both moduli with the increase of inulin concentration was determined in the study of [29]. According to the authors, increase of the moduli values may be linked with the thickening properties of polysaccharides. The effect may be result of the increasing of total solids content or the occurrence of oligosaccharide and polysaccharide interactions. However, as in the case of flow curves, the change was gradual in the range between 2.5 and 7.5% and sharp in the range from 7.5 to 10%. According to the authors, the sharp change in the range over 7.5% stems from the appearance of conditions reported for shear-induced gelation of inulin in these systems.

Table 3 presents the parameters of the power-law equations describing the relationship of the storage modulus and losses and the angular frequency, whereas Figure 3(c) presents the *K*′ and *K*^″^ dependency on the contribution of inulin. The *K*′ and *K*^″^ coefficients decreased with the increase of inulin concentration in samples. Both coefficients decreased irregularly; at the initial samples, the decrease was minor, then considerably higher, then it stabilized again, and at the highest values of inulin in the samples, the difference between values of the coefficient was highest. The *n*′ coefficient increased sharply with the increasing of inulin contribution, whereas *n*^″^ decreased in the same manner (Figure 3(d), Table 3). Based on the one-factor analysis of variance, it was determined that the means differed statistically. Relationships of the analysed values behaved in the function of inulin concentration similarly to the parameters of the power-law model used to describe flow curves. Keenan et al. [36] determined increase of *K*′ values by examining the influence of fat substitution with inulin GR and HP. As stated by the authors, the possible causes are the inulin-inulin interactions mentioned earlier and higher capacity to bind water by inulin, which leads to the increase of the moduli values [4, 37]. In the study of Juszczak et al. [4], the influence of inulin addition on the properties of gluten-free dough was analysed, and a decrease in the *K*′ and *K*^″^ values was obtained; however, it should be underlined that due to the nature of the study, the amount of water was modified, and the decrease of the value of the coefficients depended on the length of inulin chains. In turn, the study of Peressini and Sensidoni [37] examined mixtures of wheat starch and inulin. In the majority of cases, an increase of moduli values was obtained; however, the amount of water was modified as in the previously mentioned study. However, these authors verified the values at a constant amount of water in two cases. In one case, they were able to obtain an increase of *G*′ value, and in the second, a decrease along with increased amount of inulin.

It should therefore be concluded that the addition of inulin causes a decrease of the moduli value, with the exception of the cases where the content of dry matter increases, the structure produced by the replaced components is weak, or the conditions of the inulin gel production are optimal, while local limitations in the decrease in the value of moduli result from inulin-inulin and starch-inulin interactions.

**Figure 4:**
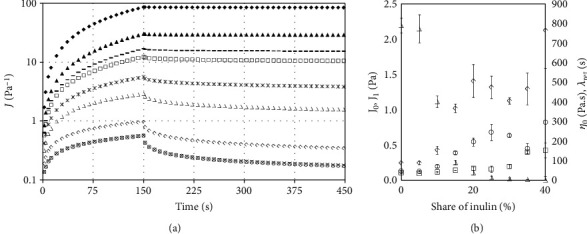
Creep and recovery test. (a) Experimental curves: ◯ 0%, × 5%, ◊ 10%, ∆ 15%, ∗ 20%, □ 25%, − 30%, ▲ 35%, and ♦ 40%. (b) Dependency of Burger's model parameters on inulin contribution: ◯ *J*_0_, ◊ *J*_1_, ∆ *η*_0_, □ *λ*_ret_.

Curves obtained in creep and recovery test are presented in Figure 4(a). In all cases, the shapes were characteristic of viscoelastic materials. Such results are typically described with mechanical models, of which the Burger's model is most commonly applied. Parameters of this model are presented in Table 4, and the dependency of the selected model parameters on the contribution of inulin is shown Figure 4(b). The lowest values of instantaneous and retardation compliance were obtained for sample without addition of inulin. With the increase of inulin concentration in the tested samples, instantaneous and retardation compliance increased, which indicates structure weakening, decrease of the share of elastic properties, and reduction of the system's resistance to deformations. The presented results differ from the study of [10], where the increase of the amount of inulin replacing sugar and galactooligosaccharides or modified starch and galactooligosaccharides resulted in a decrease of the *J*_0_ and *J*_1_ values. On the other hand, in the study of [4], increase of instantaneous and retardation compliance was obtained for inulin with low and medium DP and variable influence for inulin with high DP. The authors examined the influence of inulin on the properties of gluten-free dough, and in the case of long-chain inulin, a minor increase of *J*_0_ and *J*_1_ was observed up to 8% inulin addition and a decrease with 12% addition (below the value for control sample). Results of the our study and the presented literature data suggest that changes of the values of the analysed parameters are largely linked to the effect with other components present in the system as well as with water availability. The retardation time increased; thus, it can be concluded that inulin addition influences to the increase of the viscous properties. Similar increase of the retardation time was obtained in the study of [10]. Zero shear viscosity decreased with the increase of inulin concentration. For two first samples, a minor decrease was observed; in the subsequent ones, the difference between samples was greater. Figure 4(a) shows that for samples with 0% and 5% inulin addition, the curves do not demonstrate any differences. On the other hand, Mansouripour et al. [10] obtained an increase of the parameter. Similarly, in the study of Juszczak et al. [4], inulin with high DP resulted in an increase of zero shear viscosity, yet inulin with medium and low value led to reduction of the value of the parameter.

## 4. Conclusion

With the increasing inulin concentration, an increasingly pronounced rise of viscous properties was observed; inulin retains the liquid phase at the same time inhibiting starch pasting. The parameters of equations used to describe flow curves decreased with the increasing inulin addition. The consistency coefficient from the power model decreased with an increase in the proportion of inulin at both tested temperatures (25°C and 50°C), both for curves with increasing and decreasing shear rate. An exception was a sample with 10% of inulin at 50°C for which an increase in value was found, and a maximum value was obtained for this curve. However, the flow behaviour index behaved differently, depending on the concentration, decreasing and increasing alternately. For all samples with increasing inulin addition, the *G*′ and *G*^″^ values characterizing the tested gels were reduced, and the *G*^″^/*G*′ ratio increased, and a shift in the properties of pastes and gels towards higher viscosity was observed. In all cases, the value of retardation susceptibility increased and zero shear viscosity decreased, while instantaneous compliance and retardation time initially increased and then decreased depending on the contribution of inulin. Statistical analyses demonstrated the significance of the influence of inulin addition on the properties of pastes and gels. This proves that with addition of inulin, it is necessary to take into account its interactions with other components, so as to reduce the potentially negative changes in the product. Numerous factors must be taken into account when considering the possibilities for supplementation of inulin. If inulin is added, both the dry weight and on the other competition for water are increased. In a water deficient system, inulin may lead to the strengthening of the structure without weakening the starch structure or disturb the structure of gel formed by starch leading to its weakening. In systems, where inulin replaces ingredients, one should take into account the strength and rheological properties of these ingredients, as well as the potential weakening of the structure in the case of lack of interactions with inulin resulting from the loss of ingredient of significance for structure (e.g., starch). Properties of inulin itself are in turn strongly dependent of the process conditions; here, one should take into consideration temperature (which enables obtaining of inulin gel), as well as time necessary for structure building, the value of which may stem from interactions with other ingredients, and it may be subject to significant changes depending on the composition. Multicomponent systems may have variable impact on the process of crystallization and formation of three-dimensional network by inulin, playing both positive and negative roles in the process.

## Figures and Tables

**Table 1:** Parameters of the power-law functions describing flow properties of potato starch with addition of inulin at 50°C (mean values from two replications ± standard deviation).

S	I	Up			Down			
		*K*	*n*	*r* ^2^>	*K*	*n*	*r* ^2^>	Area
%	%	Pa s^*n*^	—	—	Pa s^*n*^	—	—	Pa s^−1^
100	0	3.20 ± 0.32^de^	0.648 ± 0.014^abc^	0.9996	6.06 ± 1.61^e^	0.530 ± 0.044^a^	0.9998	−927.2 ± 438.2
95	5	3.19 ± 0.62^de^	0.642 ± 0.023^ab^	0.9998	5.85 ± 0.17^e^	0.525 ± 0.007^a^	0.9999	−795.9 ± 245.1
90	10	3.40 ± 0.65^e^	0.616 ± 0.021^a^	0.9997	4.94 ± 0.30^e^	0.540 ± 0.002^ab^	0.9998	−299.3 ± 394.9
85	15	2.36 ± 0.34^cd^	0.637 ± 0.017^ab^	0.9996	3.38 ± 0.01^d^	0.554 ± 0.006^abc^	0.9999	−278.8 ± 143.7
80	20	1.82 ± 0.06^c^	0.654 ± 0.018^abc^	0.9995	2.74 ± 0.63^cd^	0.573 ± 0.029^bc^	0.9995	−148.3 ± 359.3
75	25	1.15 ± 0.27^b^	0.701 ± 0.025^d^	0.9998	2.37 ± 0.22^c^	0.562 ± 0.001^abc^	0.9999	−461.9 ± 99.8
70	30	1.17 ± 0.04^b^	0.695 ± 0.001^d^	0.9997	2.30 ± 0.19^c^	0.566 ± 0.010^abc^	0.9998	−425.2 ± 47.7
65	35	1.06 ± 0.02^b^	0.674 ± 0.002^bcd^	0.9996	1.70 ± 0.03^b^	0.582 ± 0.002^cd^	0.9998	−184.1 ± 3.2
60	40	0.72 ± 0.04^a^	0.682 ± 0.019^cd^	0.9997	1.03 ± 0.07^a^	0.610 ± 0.010^d^	0.9995	−43.2 ± 20.6
One-way ANOVA-*p*		<0.01	0.01	—	<0.01	0.01	—	0.08

Differences between values with same letters in particular columns are nonsignificant at 0.05 level of confidence.

**Table 2:** Parameters of the power-law functions describing flow properties of potato starch with addition of inulin at 25°C (mean values from two replications ± standard deviation).

S	I	Up			Down			
		*K*	*n*	*r* ^2^>	*K*	*n*	*r* ^2^>	Area
%	%	Pa s^*n*^	—	—	Pa s^*n*^	—	—	Pa s^−1^
100	0	10.67 ± 2.70^g^	0.534 ± 0.036^a^	0.9990	10.64 ± 1.11^g^	0.529 ± 0.004^a^	0.9999	314.6 ± 39.6^ab^
95	5	9.74 ± 1.97^fg^	0.537 ± 0.032^a^	0.9997	9.02 ± 0.23^f^	0.542 ± 0.001^b^	0.9997	868.4 ± 541.1^c^
90	10	7.11 ± 0.05^ef^	0.570 ± 0.001^abc^	0.9996	7.59 ± 0.09^e^	0.551 ± 0.003^bc^	0.9997	462.7 ± 69.4^bc^
85	15	6.23 ± 1.10^de^	0.557 ± 0.029^ab^	0.9990	6.03 ± 0.32^d^	0.554 ± 0.002^c^	0.9997	511.2 ± 240.7^bc^
80	20	5.24 ± 0.44^d^	0.569 ± 0.003^abc^	0.9993	5.36 ± 0.28^d^	0.556 ± 0.005^c^	0.9997	452.3 ± 123.9^bc^
75	25	3.52 ± 0.10^c^	0.580 ± 0.011^abc^	0.9987	3.52 ± 0.15^c^	0.572 ± 0.002^d^	0.9997	351.7 ± 104.6^bc^
70	30	2.70 ± 0.40^b^	0.626 ± 0.021^d^	0.9991	3.35 ± 0.45^c^	0.578 ± 0.012^d^	0.9996	39.5 ± 199.6^a^
65	35	2.41 ± 0.08^b^	0.592 ± 0.008^bc^	0.9997	2.17 ± 0.08^b^	0.603 ± 0.006^e^	0.9994	329.8 ± 82.9^b^
60	40	1.55 ± 0.10^a^	0.601 ± 0.014^cd^	0.9998	1.23 ± 0.06^a^	0.639 ± 0.009^f^	0.9995	248.1 ± 35.2^ab^
One-way ANOVA-*p*		<0.01	<0.01		<0.01	<0.01		0.03

Differences between values with the same letters in particular columns are nonsignificant at 0.05 level of confidence.

**Table 3:** Parameters of the power-law functions describing dependence of storage and loss moduli on angular frequency (mean values from two replications ± standard deviation).

S	I	*K*′	*n*′	*r* ^2^	*K* ^″^	*n* ^″^	*r* ^2^>
%	%	Pa s^*n*′^	—	—	Pa s^*n*″^	—	—
100	0	9.98 ± 1.03^e^	0.249 ± 0.013^a^	0.9968	4.11 ± 0.39^f^	0.327 ± 0.008^cd^	0.9982
95	5	9.17 ± 0.77^e^	0.263 ± 0.002^ab^	0.9976	4.05 ± 0.36^f^	0.320 ± 0.012^c^	0.9988
90	10	6.99 ± 0.97^d^	0.284 ± 0.012^bc^	0.9876	3.50 ± 0.35^e^	0.323 ± 0.008^c^	0.9982
85	15	4.45 ± 0.26^c^	0.311 ± 0.009^d^	0.9837	2.75 ± 0.12^d^	0.334 ± 0.002^d^	0.9978
80	20	4.05 ± 0.22^c^	0.308 ± 0.014^cd^	0.9821	2.67 ± 0.01^cd^	0.325 ± 0.007^cd^	0.9930
75	25	2.93 ± 0.32^b^	0.345 ± 0.010^e^	0.9635	2.47 ± 0.13^c^	0.319 ± 0.009^c^	0.9750
70	30	2.76 ± 0.28^b^	0.356 ± 0.007^e^	0.9677	2.42 ± 0.12^bc^	0.300 ± 0.004^b^	0.9950
65	35	2.59 ± 0.13^b^	0.312 ± 0.029^d^	0.9789	2.25 ± 0.14^ab^	0.301 ± 0.004^b^	0.9884
60	40	1.57 ± 0.28^a^	0.425 ± 0.012^f^	0.9253	2.09 ± 0.07^a^	0.285 ± 0.002^a^	0.9675
One-way ANOVA-*p*		<0.01	<0.01		<0.01	<0.01	

Differences between values with the same letters in particular columns are nonsignificant at 0.05 level of confidence.

**Table 4:** Parameters of Burger's model of potato starch gel with addition of inulin (mean values from two replications ± standard deviation).

S	I	*J*_0_	*η*_0_	*J*_1_	*λ*_ret_	*r* ^2^>
%	%	Pa^−1^	Pa s	Pa^−1^	s	—
100	0	0.134 ± 0.003^a^	790.6 ± 37.8^g^	0.247 ± 0.002^a^	38.1 ± 2.8^ab^	0.9824
95	5	0.131 ± 0.011^a^	773.2 ± 71.2^g^	0.248 ± 0.022^a^	35.1 ± 0.6^a^	0.9823
90	10	0.199 ± 0.02^b^	402.2 ± 29.9^f^	0.430 ± 0.057^b^	43.0 ± 3.0^b^	0.9836
85	15	0.391 ± 0.017^c^	92.0 ± 10.5^e^	1.023 ± 0.060^c^	52.7 ± 0.6^c^	0.9854
80	20	0.546 ± 0.058^c^	39.8 ± 11.4^d^	1.409 ± 0.233^cd^	60.9 ± 8.9^cd^	0.9886
75	25	0.682 ± 0.117^c^	15.6 ± 6.9^c^	1.321 ± 0.159^cd^	58.0 ± 15.6^cd^	0.9965
70	30	0.634 ± 0.023^c^	10.3 ± 3.4^c^	1.129 ± 0.045^cd^	71.8 ± 8.6^d^	0.9991
65	35	0.451 ± 0.073^c^	5.2 ± 0.4^b^	1.299 ± 0.226^cd^	143.4 ± 7.9^e^	0.9998
60	40	0.824 ± 0.765^c^	2.1 ± 1.0^a^	2.128 ± 0.990^d^	154.2 ± 38.8^e^	0.9999
One-way ANOVA-*p*		<0.01	<0.01	<0.01	<0.01	

Differences between values with the same letters in particular columns are nonsignificant at 0.05 level of confidence.

## Data Availability

Data will be provided upon request submitted to the corresponding author.
